# Comprehensive epidemiological profiling of poultry-derived *Salmonella* spp. in Shandong, China, 2019–2022: a longitudinal study of prevalence, antibiotic resistances, virulence factors and molecular characteristics

**DOI:** 10.3389/fmicb.2025.1541084

**Published:** 2025-03-05

**Authors:** Lele Chen, Yuxia Shi, Minge Wang, Yubao Li, Zhenshu Si

**Affiliations:** ^1^College of Agriculture and Biology, Liaocheng University, Liaocheng, China; ^2^Phage Research Center, Liaocheng University, Liaocheng, China

**Keywords:** *Salmonella*, serotype, antimicrobial resistance genes, virulence genes, poultry

## Abstract

*Salmonella* spp., as a major foodborne pathogen, pose significant threats to public health globally and has been an important zoonotic contamination for poultry industry that should receive increasing attentions. This study aimed to comprehensively investigate the prevalence, antimicrobial resistances, virulence factors, and plasmid types of *Salmonella* isolates collected from chickens, ducks, and geese across eight cities in Shandong between 2019 and 2022. Out of 300 samples, 53 *Salmonella* strains (17.67%) were isolated, with varied prevalence from 8.33% to 25.00% in different cities of Shandong. A total of seven serotypes were identified among the 53 *Salmonella* isolates, wherein the *S.* Enteritidis (45.28%), *S.* Pullorum (22.64%) and *S.* Typhimurium (16.98%) were identified as the most prevalent. Whole-genome sequencing analysis revealed that ST11, ST92, and ST19 were the predominant sequence types for *S.* Enteritidis, *S.* Pullorum, and *S.* Typhimurium, respectively. Phylogenetic analysis indicated that potential clonal spread of *S.* Enteritidis, *S.* Pullorum, and *S.* Typhimurium occurred across different regions, particularly the evidences supported that the *S.* Typhimurium isolates were dispersed in a cross-species manner. Finally, the phenotypic and genotypic profiling of antibiotic resistance among the isolates revealed that these isolates were multidrug resistant with corresponding antibiotic resistance genes (ARGs) including *bla*_TEM_, *aac*, *aph*, *tet*(A), and *tet*(B) to confer them with resistances to commonly-used veterinary drugs such as *β*-lactams, quinolones, macrolides. To sum, this study provides valuable insights into the current epidemiology of *Salmonella* in poultry industry in one of the biggest provinces in China, and shedding the light on the urgent necessity for further approaches to prevent and decontaminate such MDR *Salmonella* in livestock under One Health concept.

## 1 Introduction

*Salmonella* spp., as one of the most prevalent foodborne pathogen, are reportedly to cause severe and acute intestinal diseases called salmonellosis via contaminated food chain (Chu et al., 2024). The previous studies indicated that *Salmonella* spp. are versatile pathogens capable of infecting a wide range of host animals, including chickens, cattle, and pigs, which are closely related to anthropogenic environments and humans. The authorities in the United States estimated that *Salmonella* causes approximately 1.35 million cases of infections with 420 deaths reports annually (Centers for Disease Control and Prevention | CDC). It is noted that the poultry has been a major source and an important reservoir of *Salmonella* spp. (Andoh et al., 2017; Feasey et al., 2012; Pui et al., 2011). Contaminated poultry products such as meat and eggs, are common cues for *Salmonella* accumulation and subsequent salmonellosis in humans (Lamas et al., 2016; Tedersoo et al., 2022). A directly evidence is the large foodborne infection outbreak across the U.S. in 2018 had been linked to raw chicken meat, highlighting the widespread prevalence of *Salmonella* in the broiler industry serves as a health threat that should not be neglected (Chu et al., 2023). Additionally, *Salmonella* spp. demonstrated high adaptive tolerance in different harsh conditions to transmit from animals and environments to related products through fecal contamination (Smith et al., 2023). A prior study on free-ranged poultry revealed that *Salmonella* was able to transmit from poultry to workers within in the same ecology via daily contacts. Therefore, longitudinal epidemiological analysis of *Salmonella* in poultry is crucial for in-depth understanding toward prevalence and genomic insights, which helps to better control on their potential outbreak.

Currently, antibiotics like fluoroquinolones and cephalosporin are frequently used to eradicate *Salmonella* infections (Chiu et al., 2002). However, the misuse and overuse of such antibiotics in animals have led to a global increase of antibiotic resistance in *Salmonella* spp. dampening the efficacies of such agents for clinical treatment against *Salmonella* (Wang et al., 2021; Xu et al., 2020). Previous studies have shown that infections caused by antibiotic-resistant bacteria (ARB) greatly challenge the current treatment paradigm and are associated with higher mortality rates in clinical settings (Crump et al., 2008). Moreover, it has been confirmed that both farm animals and wildlife are able to carry the identical *Salmonella* serotypes, thereby leading to high level dispersion of such ARB among the different host in the same niches (Cui et al., 2019).

Innovations in genomic analysis contribute accumulative genomic data of *Salmonella* in the public database, which generated from cost-effective, high-throughput whole-genome sequencing (WGS) (Wang et al., 2023). WGS is progressively becoming the standard approach for in depth understanding of pathogens. As a foundational tool in current microbiology studies, WGS data also facilitate the identification of virulence factors and antimicrobial resistance (AMR) genes (Chiou et al., 2023; Chiou et al., 2022; Collineau et al., 2019; Sohail et al., 2021). Notably, *Salmonella* Pathogenic Islands (SPIs) that house *Salmonella* virulence genes play a significant role in the pathogenesis of *Salmonella* in divergent hosts (Hensel, 2004; Rychlik et al., 2009). The previous investigations highlighted that SPIs vary among the serotypes and may explain differences in virulence across *Salmonella* serotypes (Zhao et al., 2020). Therefore, the surveillance of these ARGs and virulence factors are crucial for comprehending the evolution and pathogenicity of *Salmonella*.

In this study, we conducted a longitudinal study on the prevalence of *Salmonella* spp. in chicken, duck, and goose populations across eight cities in Shandong, China from 2019 to 2022. Subsequently, we phenotypically and genotypically identified the antimicrobial resistance of collected *Salmonella* strains by analyzing MLST, phylogenetic relationships, antibiotic resistance genes (ARGs), and virulence factors. This study provides vital information into the molecular epidemiology and potential pathogenicity of poultry-derived *Salmonella*, and contributes to development of timely control before the spread of such concerning pathogens.

## 2 Materials and methods

### 2.1 Sample collection

A total of 300 fecal samples were collected from the chickens (*n* = 225), ducks (*n* = 67), and geese (*n* = 8) from eight regions in Shandong, China, including Liaocheng, Weifang, Yantai, Qingdao, Zibo, Heze, Jinan, and Dongying, during 2019 to 2022. The sampling was conducted with the informed consent of the poultry farms. The samples were labeled, placed in sterile plastic sample bags, transported to the laboratory on ice, and processed promptly.

### 2.2 Isolation and serotype identification of *Salmonella*

The collected samples were inoculated onto *Salmonella*-Shigella (SS) selective agar, where *Salmonella* colonies manifest as black, pale yellow, or colorless on the SS agar medium (Maddocks et al., 2002). Presumptive *Salmonella* isolates underwent PCR examination targeting the *invA* and *ompC* genes, with the *Salmonella* Abony NCTC 6017 as a positive control (Chu et al., 2023). The serotype of the *Salmonella* isolates was determined using the Kauffmann-White scheme via slide agglutination with commercial O and H antisera (DK-2300 CPH.S Denmark). All *Salmonella* isolates were preserved as 50% glycerol stocks in a − 80°C freezer. Confirmed *Salmonella* isolates were serotyped according to the Kauffmann-White-Le Minor’s scheme (Guibourdenche et al., 2010).

### 2.3 Antimicrobial susceptibility test (AST)

All *Salmonella* isolates were subjected to the test for 15 antibiotics and the results were interpreted using the Kirby-Bauer disk diffusion method for antimicrobial susceptibility (https://www.addl.purdue.edu/newsletters/1997/spring/dds.shtml) on commercial antimicrobial disks (Hangzhou Microbial Reagent Co., Ltd. Hangzhou, Zhejiang, China) following the guidelines established by the Clinical Laboratory Standards Institute (CLSI: https://clsi.org/) (Humphries et al., 2021). Based on CLSI zone diameter interpretation criteria each strain was categorized as resistant, intermediate, or susceptible. Strains displaying resistance to a minimum of three different antibiotic classes, excluding cross-resistance mechanisms, were classified as multidrug-resistant (MDR) (Wang et al., 2023). The tested antibiotics included: amikacin, tobramycin, neomycin, doxycycline, azithromycin, lincomycin, polymyxin, amoxicillin, clindamycin, imipenem, spectinomycin, levofloxacin, enrofloxacin, cefotaxime, and florfenicol. The AST was quality controlled by using *Escherichia coli* ATCC 25922 as the reference strain.

### 2.4 WGS analysis

**Figure 1:**
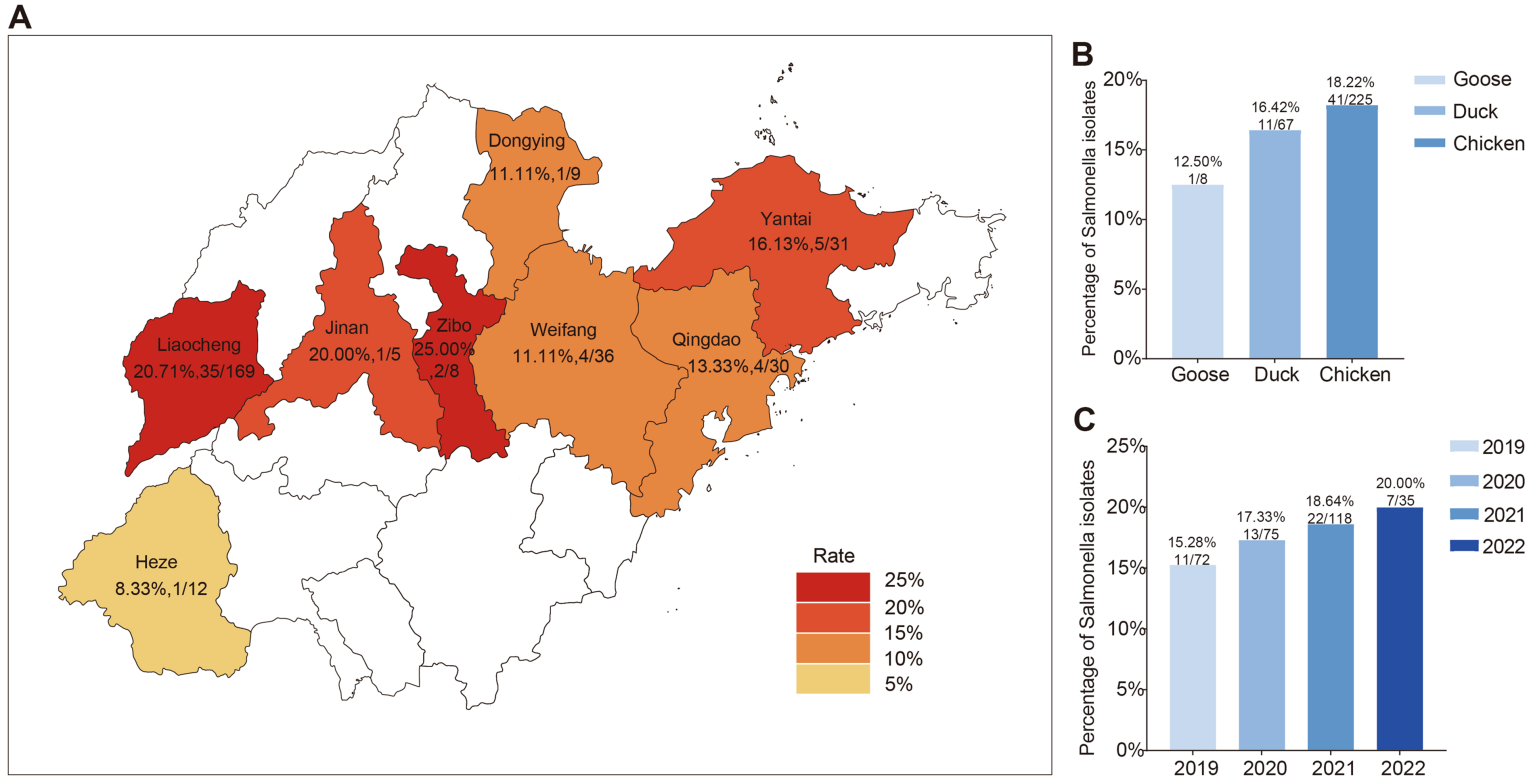
Geographic distribution and epidemiological overview of *Salmonella* in Shandong province. **(A)** Map of *Salmonella* sampling areas in Shandong province. **(B)** Contamination rates of *Salmonella* Isolates across various hosts. **(C)** Isolation rates of *Salmonella* Isolates over different years.

All *Salmonella* isolates were subjected to the WGS after extraction of genomic DNA using the commercial kit (TIANGEN, China) following the manufacturer’s instructions. The purity and concentration of DNA were assessed with a Nanodrop 2000c spectrophotometer. WGS was carried out using the Illumina Hi Seq 2,500 system (Novo gene, Guangzhou, China) with a paired-end 2 × 150 bp sequencing protocol. All data were filtered to remove adaptors and low-quality reads using fastp v0.23.4 (fastp -i in.R1.fq.gz -I in.R2.fq.gz -o out.R1.fq.gz -O out.R2.fq.gz) (Chen et al., 2018b) and Fastqc v0.12.1 (fastqc *.fq.gz) (https://ifbic05c871deb3994101hwc60ck5ncovf6xf9fiac.eds.tju.edu.cn). Draft genomes were *de novo* assembled using SPAdes version 3.13.1. Multi-locus sequence typing (MLST) analysis was carried out using the online tool available at https://github.com/tseemann/mlst. Virulence genes, plasmid incompatibility (Inc) groups, and antibiotic resistance genes (ARGs) were analyzed using the online toolkits at https://github.com/tseemann/abricate. Phylogenetic trees for the *Salmonella* isolates were constructed using CSI Phylogeny version from https://www.genomicepidemiology.org/. When utilizing all the aforementioned online tools, default parameters were applied (Figure 1).

## 3 Results

### 3.1 Prevalence and distribution of *Salmonella* spp. in poultry from Shandong

**Figure 2:**
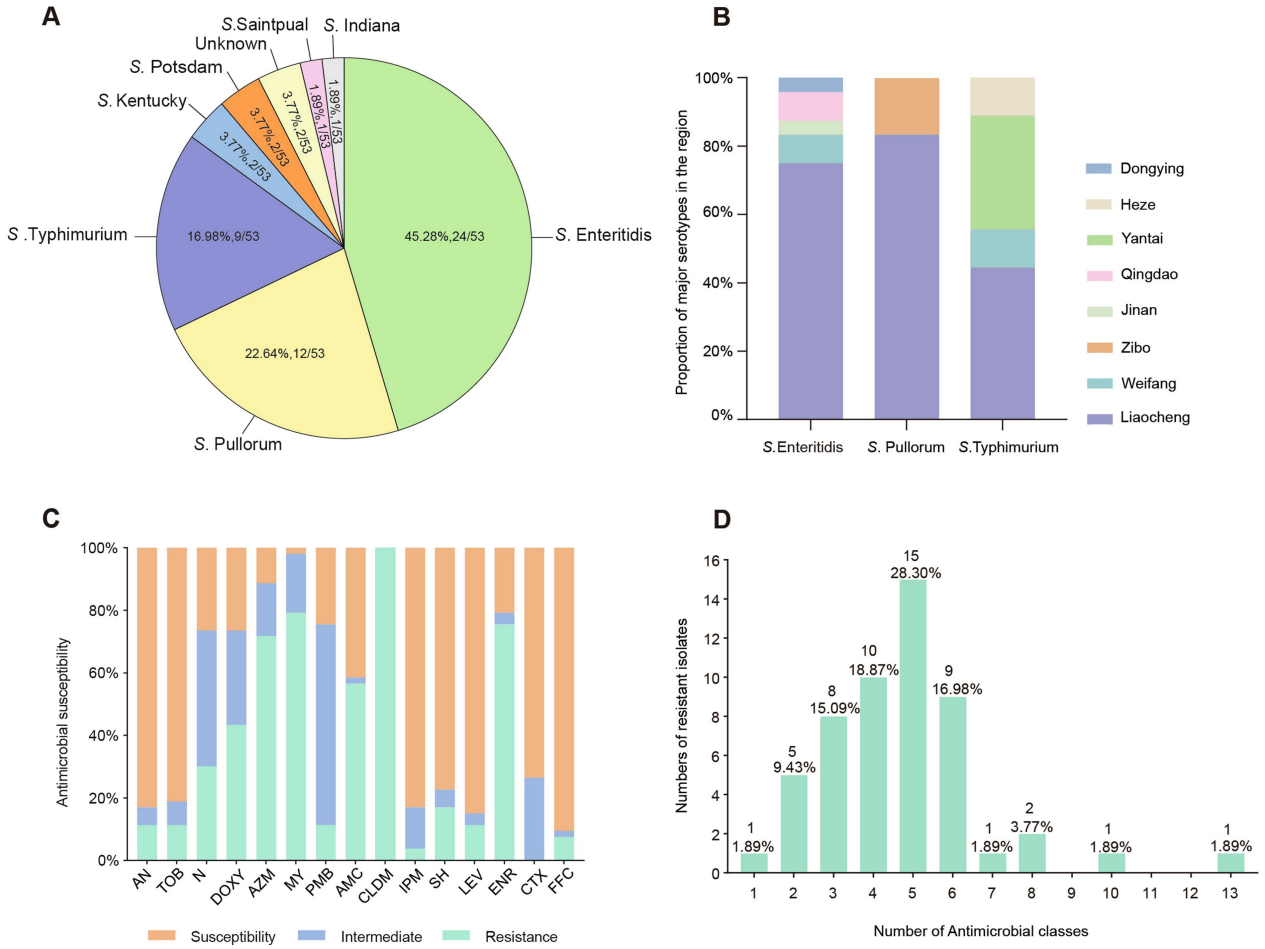
The serotypes and resistance phenotype of 53 *Salmonella* isolates. **(A)** The serotype identification of *Salmonella*. **(B)** Serotype distribution of *Salmonella* in different regions. **(C)** Susceptibility of *Salmonella* to various antimicrobial agents. **(D)** Numbers of resistant *Salmonella* isolates.

In this study, a total of 300 samples encompassing 225 from chickens, 67 from ducks, and 8 from geese, where 53 *Salmonella* isolates (17.67%, 53/300) were collected across eight cities in Shandong province between 2019 and 2022. The regional distribution of *Salmonella* isolates was shown as follows: Liaocheng (20.71%, 35/169); Weifang (11.11%, 4/35); Yantai (16.13%, 5/31); Qingdao (13.33%, 4/30); Zibo (25.00%, 2/8); Heze (8.33%, 1/12); Jinan (20.00%, 1/5); Dongying (11.11%, 1/9) (Figure 1A). As depicted in Figure 1B, the isolation rate in chickens was 18.22% (41/225), which was the highest among poultry species, including chickens, ducks, and geese. The temporal dynamic of the *Salmonella* prevalence numerically increased in first 3 years (11, 13, 22 respectively) then followed a slight decrease in 2022. However, the prevalence rate of *Salmonella* isolates in 2022 (20.00%, 7/35) was significantly higher compared to 2019 (15.28%, 11/72), suggesting a putative increase in salmonellosis incidence in poultry farms in Shandong during 2019–2022 (Figure 1C). A total of 7 serotypes were identified among the 53 *Salmonella* isolates, yet 2 isolates were not successfully determined (Figure 2A). The most prevalent serotype was *S*. Enteritidis (45.28%, 24/53), followed by *S*. Pullorum (22.64%, 12/53), *S*. Typhimurium (16.98%, 9/53), *S*. Kentucky (3.77%, 2/53), *S*. Saintpaul (1.89%, 1/53), and others. It was of note that the chicken samples from Liaocheng predominantly carried the isolates belonging to *S*. Enteritidis and *S*. Pullorum (Figure 2B).

### 3.2 Antibiotics susceptibility test (AST) of *Salmonella* isolates

In this study, all 53 *Salmonella* isolates were subjected to the AST with 15 commonly-used antibiotics. As depicted in Figure 2C, the all of isolates demonstrated resistance to clindamycin (100.00%, 53/53), and majority of them were found to resistant to lincomycin (79.25%, 42/53), enrofloxacin (75.47%, 40/53), and azithromycin (71.70%, 38/53). In addition, certain strains exhibited moderate resistance to azithromycin (64.15%, 34/53). Conversely, certain isolates showed low-level resistance to imipenem (100.00%, 53/53), florfenicol (90.57%, 48/53), spectinomycin (84.91%, 45/53), amikacin (83.02%, 44/53), tobramycin (81.13%, 43/53). Notably, the enrofloxacin resistance rate (100.00%) of *Salmonella* isolates in 2022 was significantly higher compared to the samples collected in years before. Moreover, a large portion of *Salmonella* isolates exhibited a multi-resistant profile, showcasing resistance to multiple antibiotics. Specifically, 88.68% (47/53) of the strains demonstrated resistance to three or more antibiotics, with an alarming strain S9 displaying resistance to 13 antibiotics (Figure 2D).

### 3.3 Molecular characteristics of *Salmonella* isolates

**Figure 3:**
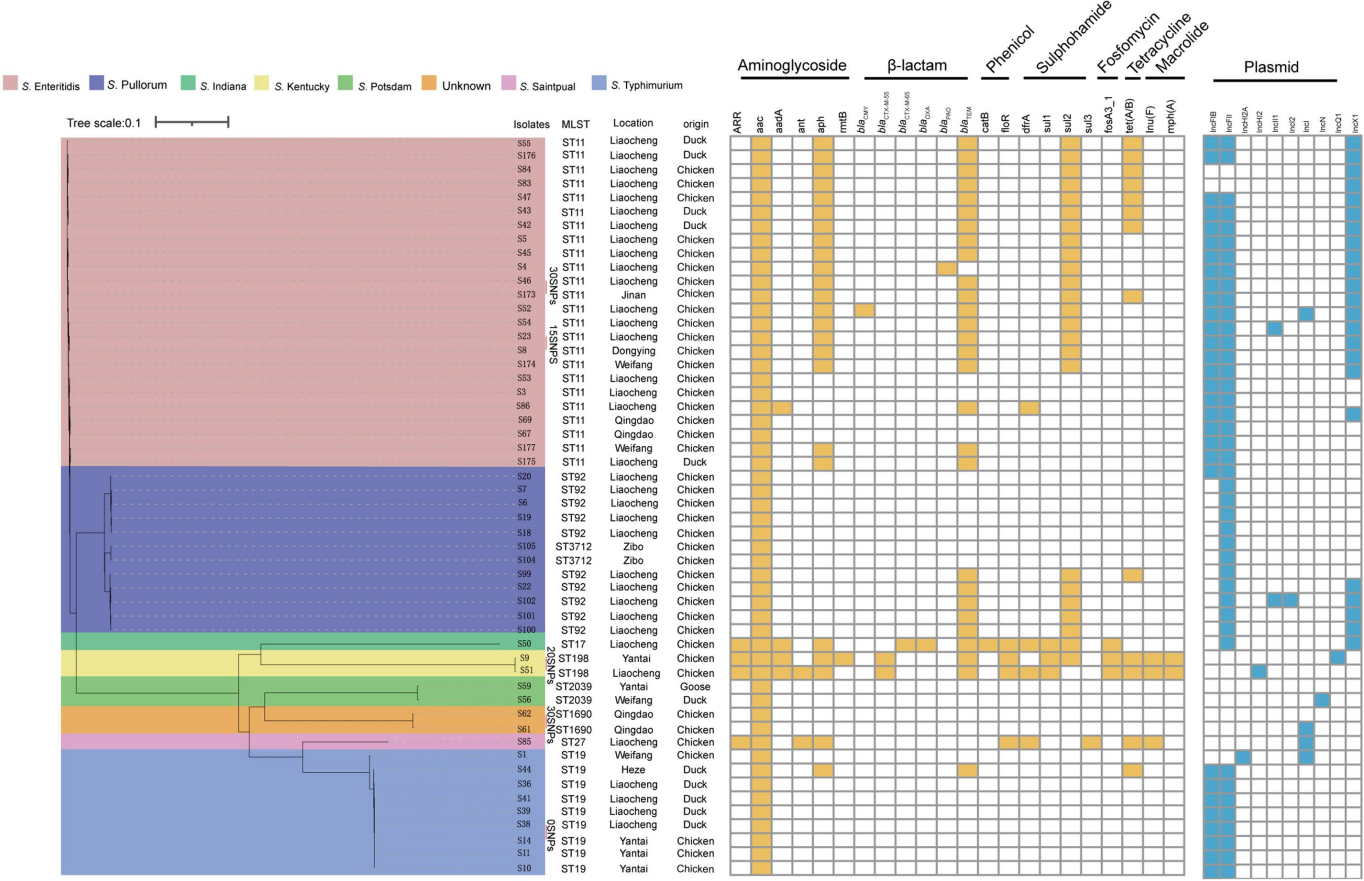
Phylogenetic structures, MLST and antibiotic resistance genotypes of 53 *Salmonella* isolates.

All *Salmonella* isolates were subjected to the WGS for harvesting the genomic insights, based on which the subsequent MLST analysis confirmed that *S. indiana, S.* Kentucky*, S.* Enteritidis, and *S.* Typhimurium were assigned to sequence types ST17, ST198, ST11, and ST19, respectively. Moreover, *S*. Pullorum isolates were categorized into two distinct STs, namely the ST92 (*n* = 10) and ST3717 (*n* = 2) (Figure 3). The core-genome sequences of *S.* Typhimurium isolates from chicken farms in Yantai (S14) were intriguingly found to be identical to the strains from duck farms in Liaocheng (S38), and a *S*. Kentucky isolate from Yantai (S9) demonstrated high similarity with an isolate from Liaocheng (S51) by showing only 20 single nucleotide polymorphisms (SNPs) in the genome. Likewise, a *S.* Enteritidis isolate from chickens in Liaocheng (S46) was phylogenetically similar to an isolate in Jinan (S173) with only SNP count of 30 (Figure 3). These findings collectively suggested that clonal spread at strain level might be a significant driving force for the dissemination of *S.* Typhimurium and *S.* Enteritidis across regions in Shandong province. Furthermore, one *S.* Enteritidis isolate (S50) exhibited more than 30,000 SNPs with many other isolates (S45, S46, S47, S5, S52, S53, S54, S83, S84, S86) from the same sampling site in Liaocheng, indicating the high genetic diversity among *S.* Enteritidis genogroups presented within the same geographical area.

### 3.4 Analysis of antibiotic resistance genes

As the AST suggested the presence of phenotypical resistances against commonly-used antibiotic, we next sought to investigate the genotypic features of the antibiotic resistances. The genomic analysis revealed that 22 different types of ARGs were detected to confer resistances to 7 classes of antibiotics (Figure 3). As one of the most clinically concerning resistance genotype, the ESBL-producing genes were frequently detected among the Salmonella isolates, where *bla*_CTX-M_ and *bla*_TEM_ were identified as most dominant subtypes with prevalence rate of 54.72% (29/53) and 52.83% (28/53) respectively, followed by with other *β*-lactam resistance genes such as, *bla*_CMY_ (1.89%, 1/53), *bla*_PAO_ (1.89%, 1/53), and *bla*_OXA_ (1.89%, 1/53). Additionally, a plenty of other clinically significant ARGs were detected, including aminoglycoside resistance genes *ARR* (7.55%), *aac* (100.00%), *aad* (7.55%), *ant* (3.77%), *aph* (45.28%), and *rmtB* (1.89%); phenicol resistance genes *floR* (7.55%) and *catB* (1.89%); macrolide resistance genes *lnu* (5.66%) and *mph* (3.77%); sulphonamide resistance genes *sul1* (5.66%), *sul2* (45.28%), and *sul3* (1.89%); trimethoprim resistance genes *dfrA* (9.43%); fosfomycin resistance gene *fosA3* (5.66%); and tetracycline resistance genes *tet*(A/B) (24.53%).

### 3.5 Presences of plasmid

**Figure 4:**
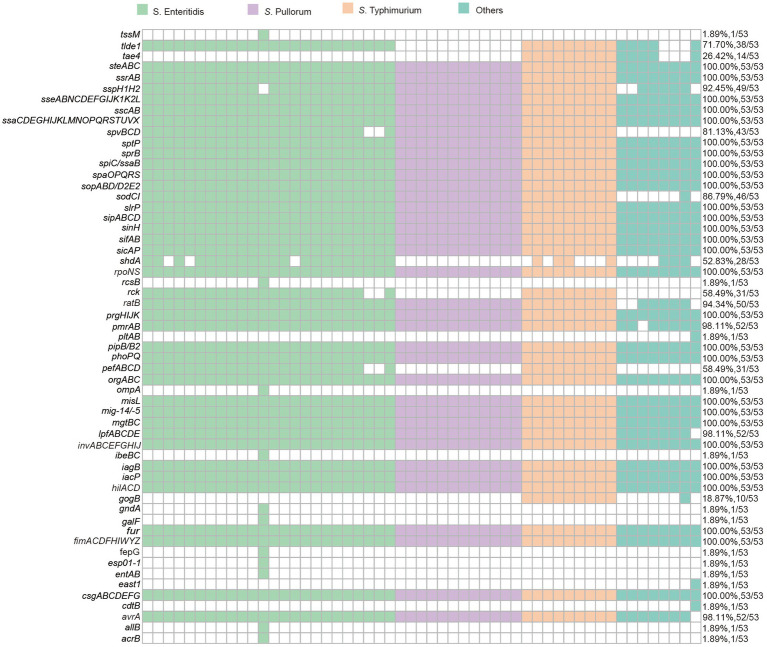
The presence of virulence factors and virulence genes in 53 *Salmonella* isolates.

In the prokaryotes, the plasmids are deemed as the most imported carrier to facilitate the ARGs transmission. Thus, we have profiled the genetic characteristics of plasmids among the isolates. The results showed that a total of 9 plasmid replicon types were identified among the *Salmonella* isolates, including IncFIB (58.49%, 31/53), IncFII (81.13%, 43/53), IncHI2 (1.89%, 1/53), IncI1 (3.77%, 2/53), IncI2 (1.89%, 1/53), IncI (7.55%,4/53), IncN (1.89%, 1/53), IncQ (1.89%, 1/53), and IncX1 (43.40%, 23/53). Of note, no plasmid replicon was detected in *S*. Kentucky isolates. Among the plasmids, IncFIB and IncFII, which are the most prevalent, are primarily concentrated in *S*. Enteritidis and *S*. Typhimurium serotypes of *Salmonella*. This suggests that the presence of these plasmids may be associated with the serotype of the *Salmonella* strains. Furthermore, IncFIB was found in seven cities other than Zibo, while IncFII was detected in all of the surveyed cities. This indicates that the types of plasmids vary across different regions (Figure 4).

### 3.6 Virulence factor

As to the harbored virulence factors, a total of 57 types of genes that responsible for *Salmonella* virulence were identified in all *Salmonella* isolates, most of which (31/57) were found to be conserved across all isolates. These virulence factors included handful genes belong to gene clusters encoding type III secretion systems (T3SS), SPI-2 effectors, and the *Salmonella* virulence plasmid *(pSV).* Notably, genes such as *spv, lpfABCDE,* and *pefABCD* were present in all isolates. Additionally, *cdtB* and *pltAB* genes were exclusively determined in the *S. indiana* isolates, in which genes like *cdtA*, *cdtC*, *mig-5/−14*, and *rck* were also found to be co-existed.

## 4 Discussion

*Salmonella* spp. is considered one of the most significant foodborne pathogens and rank as the third leading cause of human mortality related to diarrheal illnesses. Animals serve as the primary reservoir for *Salmonella*, with animal-derived foods being the essential route for the transmission to humans via food chain (Ferrari et al., 2019). This study aimed to assess the prevalence and distribution of *Salmonella* isolates from poultry species in the Shandong province, which is one of the major bases of agricultural and livestock industry in China.

From the samples collected in eight major cities in Shandong during 2019 to 2022, a total of 53 *Salmonella* isolates were identified. This isolation rate was higher than that previously reported in Xinjiang (11.0%, 8/73) and Guangdong Province (3.58%, 301/8405), yet much lower than that in Tibet (30.4%, 70/230) and Henan (48.7%, 131/269) (Perry et al., 2024). However, the exact prevalence rates of *Salmonella* were observed heterogeneous in the different sampling sites of the eight cities, ranging from 8.3 to 25% accordingly. A previous study reported *Salmonella* detection rates in 12 provinces were ranging from 3.6 to 12.9% (Gong et al., 2014), indicating the *Salmonella* prevalence might be under certain geographic influences. The study also showed a notable presence of *Salmonella* contamination in poultry in Shandong Province, particularly in chickens which was with an alarming rate of 18.2% (41/225) (Liyuan et al., 2023). These results implied a wide presence of *Salmonella* contaminations in varied poultry breeds. Moreover, the isolation rate of *Salmonella* was also reported to be varied across the different environments, as some studies evidencing that the detection of *Salmonella* increased from poultry farms (9.1%) to slaughterhouses (9.5%) and finally enriched in the retail markets (41.8%) (Dargatz et al., 2016; Papadopoulos et al., 2016; Shang et al., 2019). To conclude, these differences were under impact of many factors such as geographical factors, environmental conditions and poultry species/breeds (Cao et al., 2017).

The prior study highlighted the increasing prevalence of *Salmonella* were potentially influenced by climate changing and dietary supplementations. For example, investigation by Sonora showed that *Salmonella* isolation rates were found to elevate in concert with the increment of temperature (Davies and Breslin, 2002). This suggests that global warming and climate change may promote *Salmonella* dispersion in nature especially in the anthropogenic environments. Factors such as antibiotic use, poultry density, and environmental pollution are as well believed to contribute to the global rise of *Salmonella* prevalence (Davies and Breslin, 2002; Sohail et al., 2021). It underscores the urgent necessity to monitor the *Salmonella* spp. and the preventive measures by relevant authorities.

This study identified *S.* Enteritidis as the predominant serotype among the collected isolates, followed by *S*. Pullorum and *S.* Typhimurium. In recent years, the contamination of *S.* Enteritidis has been a critical health concern in many provinces of China (Gong et al., 2014; Yang et al., 2019). This indicates that the widespread of *S.* Enteritidis is plausibly linked to the extensive contamination of poultry and poultry products. Cases of salmonellosis due to the consumption of contaminated poultry products, including meat, eggs and fresh products, have been reported as early in 2011 (Collineau et al., 2020; Middleton et al., 2014; Varga et al., 2012). *S.* Typhimurium, one of the most common serotypes in humans, have been frequently associated with severe infections on pigs, cattle, and other livestock in the European Union and the United States (Kuus et al., 2021). However, currently, this species has spread extensively worldwide, affecting not only fresh pork and beef but also poultry meat and poultry-related products. The consumption of contaminated livestock and poultry products has been proposed as a significant driver for bacterial infections in human (Perry et al., 2019; Ploton et al., 2017; Shen et al., 2022).

The tremendous use of antibiotics in animal production to promote growth and combat infection contributes to the rapid development of antibiotic resistance among pathogens (Liyuan et al., 2023). Our study revealed high levels of resistance in *Salmonella* isolates to clindamycin, lincomycin, ampicillin, enrofloxacin, azithromycin, and doxycycline, possibly owing to large-scale antibiotic usage for poultry farming. The S9 *Salmonella* strain exhibits resistance to 15 different antibiotics, making it a typical example of multidrug-resistant (MDR). *Salmonella* strains in the late 1980s challenged treatment choices amoxicillin. The MDR strains of *Salmonella* exhibit various resistance pattern, including ACSSuT (ampicillin, chloramphenicol, streptomycin, sulfamethoxazole, tetracycline) and AKSSuT (ampicillin, kanamycin, streptomycin, sulfamethoxazole, tetracycline) resistance pattern has been commonly reported. And this often includes first-line antibiotics (*β*-lactamases, macrolides), and the rise of MDR strains has compelled clinicians to resort to second-line agents (quinolones, tetracyclines and third-generation cephalosporins) (Rahman et al., 2023; Ramatla et al., 2021; Rowe et al., 1997; Samia et al., 2021). It is of particular concern that β-lactams and fluoroquinolones, which are typically the primary options for treating salmonellosis, were found to be lack of potency to the strains collected in our study due to carriage of corresponding ARGs. As observed with the emergence of *Salmonella* Infantis (ESI) clone in 2014, which was first detected in retail meats in Tennessee, by 2019, it had spread across the United States. The pESI plasmid underwent significant recombination, carrying not only the extended-spectrum β-lactamase gene *bla*_CTX-M-65_, but also the *gyrA* mutation, which confers resistance to fluoroquinolones, further limiting treatment options (Perry et al., 2024). Additionally, *Salmonella* strains are often transmitted through contaminated food, wastewater, animals, and other sources (Cosby et al., 2019; Gebreyes et al., 2008). This suggests that MDR strains have the potential for spread, which undoubtedly poses a significant threat to public health, exacerbates the burden on clinical medicine, and imposes a tremendous economic strain on society. However, the resistances to cephalosporins and fluoroquinolones have been observed to increase in many countries, possibly due to incorrect antibiotic usage. Additionally, Shea (2004) highlighted that prolonged antibiotic therapy contributed to development of antimicrobial resistance. Ciprofloxacin and enrofloxacin-resistant *Salmonella* isolates have been widely found in aquaculture, poultry, poultry meat, and pig manure (Ubeyratne et al., 2023; Wang et al., 2022), which threaten both animal wellbeing and human health under One Health concept.

MLST analysis revealed that all *S.* Enteritidis, *S.* Typhimurium, *S*. Kentucky, and *S. indiana* isolates belonging to ST11, ST19, ST198, and ST17, respectively. ST11 emerged as a predominant serotype, particularly prevalent chicken samples in our cases. Notably, 95% of *S.* Enteritidis isolates in England and Wales from April 2014 to March 2015 were classified as ST11 (Aung et al., 2022). The European Food Safety Authority (EFSA) indicated that the outbreak of infection of *Salmonella* ST11 in 2022 in Europe was associated with eggs and egg products, underscoring the urgent need for controlling the *Salmonella* contamination in poultry and its supply chain. The ST19 is the predominant genotype of *Salmonella* isolate with broad geographic distribution (Achtman et al., 2012; Gómez-Baltazar et al., 2023). The ST19 *Salmonella* is commonly found in patients with diarrhea and is always detected in various origins including animals, food, and humans (Sun et al., 2014; Wong et al., 2013). *S*. Kentucky, an emerging pathogen, has been increasingly responsible for human non-typhoidal *Salmonella* infections since 2005 (Mahindroo et al., 2019). While *S*. Kentucky isolates are frequently found in poultry and livestock yet rarely associated with human infection. Furthermore, the ST198 has been observed to spread independently among poultry and other livestock, occasionally leading to human infections (Samper-Cativiela et al., 2022). In this study, *S*. Pullorum was found with ST92 and ST3717.

Phylogenetic analyses are crucial for understanding microbial evolution and infectious disease transmission. Bacterial phylogenies are often inferred from SNP alignments, with SNPs as the method in the genetic relationship between isolates, allowing for comparison of genetic information at the genome level for each isolate (Dallman et al., 2018; Mona et al., 2025). In this study, the core-genome sequences of *S*. Kentucky isolate from Yantai (S9) demonstrated high similarity with an isolate from Liaocheng (S51) by showing only 20 single nucleotide polymorphisms (SNPs) in the genome. The genetic distance between these isolates is small, suggesting that they may originate from the same clonal group, which is consistent with previous *Salmonella* studies. Specifically, isolates with a small SNP distance are highly likely to come from the same clonal lineage (Wang et al., 2018). Additionally, SNP analysis revealed significant genetic diversity among these isolates. For example, isolate S50 showed over 30,000 SNP differences compared to several other isolates from the same sampling site (Liaocheng), including S45, S46, S47, S5, S52, S53, S54, S83, S84, and S86. This finding suggests that the genetic variation may be related to the source and time of isolation, with the primary factor being the origin of the *Salmonella* isolates (Levent et al., 2021). With this estimate of genetic similarity, bioinformaticians can better identify isolates that likely have a recent common source, and provides essential data for public health management.

The phenotypic resistances are closely associated with ARGs. Genotypic analysis revealed that the most prevalent ARG gene was *aac*, followed by *aph*, *sul2*, and *tet* (A/B). These genes conferred the resistances to aminoglycosides, sulfonamides, and tetracyclines (Ubeyratne et al., 2023). Further analyses also uncovered the presence of resistance genes against *β*-lactamase, including *bla*_TEM_, *bla*_OXA_, *bla*_CTX-M-65_, *bla*_CTX-M-55_, and *bla*_CMY_. Among them, *bla*_TEM_ was the most prevalent (Olesen et al., 2004). These β-lactamases are active to hydrolyze the cephalosporins, dampening the efficacies of such antibiotics. Notably, genes like *aadA* rendered the isolates resistant to streptomycin, gentamicin, and tobramycin (Chu et al., 2023; Samia et al., 2021). Previous studies have also identified these genes and their impact on antimicrobial resistance in *Salmonella* spp.

Plasmids have been reported as a key player in the dissemination of ARGs, virulence genes, and other traits that provide a fitness advantage to host bacteria. Analysis on plasmid in the current study revealed the presence of 9 different plasmid replicons existing in the *Salmonella* isolates, with the IncFII as the dominant replicons. These plasmids were found in various host bacteria, indicating their ability to spread the harbored genes with their transmissions. IncFII and IncFIB are the most common variants within the IncF plasmid family. They are important vectors for the carriage and transmission of antibiotic resistance and virulence genes (Kline, 1985; Ravi et al., 2017; Villa et al., 2010). They are important vectors for the transmission of antibiotic resistance and virulence genes. In particular, IncFII plasmids are also the main vector of *mcr-1* in *E. coli* isolates (Sugawara et al., 2019; Xavier et al., 2016). The IncF plasmids in Enterobacteriaceae are of particular interest since they contribute to the carriage and spread of ARGs and virulence genes (Carattoli, 2011). IncF plasmids have been isolated that contain ARGs genes. IncF plasmids in *Salmonella* isolated in China carried fluoroquinolone resistance genes (Chen et al., 2018a). In *Salmonella* isolated in the United States, IncF plasmids have been associated with *strAB*, *tetA*, *tetC*, *tetD*, *aphA* (aminoglycosides), and *sul2* (Han et al., 2012; McMillan et al., 2019; McMillan et al., 2020), and they were most frequently described carrying ESBL genes, encoding carbapenemases genes, and plasmid-mediated quinolone resistance (PMQR) genes (Rozwandowicz et al., 2018). In addition, they also carry specific virulence traits such as cytotoxins and adhesion factors as accessory genes (Johnson and Nolan, 2009). In this study, IncF family plasmids were isolated from multiple serotypes of *Salmonella*, primarily *S.* Enteritidis and *S.* Typhimurium, across different regions. This suggests that IncF family plasmids may be associated with specific regions and serotypes. These findings are in accordance with previous studies, where the IncF family was detected in multiple serotypes of *Salmonella* across different regions and farms (NCF3, −5, and − 6) (Pornsukarom and Thakur, 2017; Wang et al., 2013). The association between plasmid types and specific serotypes or geographical regions may lead to the spread of antibiotic resistance and treatment limitations, exacerbating the antibiotic resistance crisis and posing a significant threat to global public health. Furthermore, IncHI2, as part of the backbone element of the pSE380T plasmid, in conjunction with the IncFIA plasmid, produces a rare fusion product encoding both virulence and resistance genes with high transmissibility (Villa et al., 2010; Wong et al., 2017). Therefore, monitoring the prevalence of ARGs together with plasmids profiling is crucial for the prevention and control of antibiotic-resistant *Salmonella*.

Analysis of WGS data revealed that the *Salmonella* isolates were found with multiple virulence factors including *invABCEFGHIJ*, *prgHIJK*, *hilACD*, *sicAP*, *sipABCD*, *sopABD/D2E2*, *spaOPQRS*, *sptP*, and *mgtBC*. Within these, virulence genes like *invA*, *mgtC*, and *sopB* are clustered in the *Salmonella* Pathogenicity Island (SPI) on the chromosomes, help *Salmonella* to establish systematic infection by mediating cell invasion, intracellular survival, and inflammatory responses (Marcus et al., 2000). For instance, *hilACD* are able to activate transcription of key virulence genes on SPI-1, facilitating the release of essential effector proteins for invading the host intestine (Bosire et al., 2020). Furthermore, the *spv* genes located on the *Salmonella* virulence plasmid (IncF), which is involved in survival and systemic infection in host cells, were detected in *S.* Enteritidis and *S.* Typhimurium. The *spvABCD* genes are located upstream of the genes *pefA* (plasmid-encoded fimbriae) and *rck* (resistance to complement killing) in a virulence plasmid by the upstream spvR gene (Monte et al., 2020; Silva et al., 2017). However, *spvR*, which is needed for the regulation of the *spv* locus (Guiney and Fierer, 2011), was absent in our strains. Previous studies have indicated that the *spv* operon, which plays a role *Salmonella* survival within the host cell during systemic infection, affects the formation ofautophagosomes, as well as highlight its association in killing of macrophages and neutrophils (Wu et al., 2016), being crucially required for virulence *in vivo* (Passaris et al., 2018), including aggravated damage in zebrafish infection model (Cao et al., 2023; Hsu et al., 2019; Passaris et al., 2018). Moreover, other virulence factors like *pefABCD*, *lpfABCDE*, *cdtB*, *pltAB* and *rck* were detected. The *lpfABCDE* and *pefABCD* gene clusters on *pSV* are reported to regulate the formation of long polar fimbriae fimbriae. *The cdtB* is a common virulence gene found in certain Gram-negative bacteria, and the expression of *cdtB* promotes toxin production in non-typhoidal *Salmonella* (Wójcicki et al., 2021). In addition, *cdtB* was characterized in *S. typhi* as one component of the cytolethal distending toxin (CDT) (Haghjoo and Galán, 2004). Several Gram-negative bacterial pathogens, including *S. typhi*, produce CDT, which arrests growth, induces apoptosis of infected host cells, and enhances the persistence of pathogenic bacteria in the host. The *cdtB* genes in *S. typhi* is located on a pathogenicity island upstream of the *pltA* and *pltB* genes, which encode pertussis-like toxins A and B (*PltA* and *PltB*). These toxins form a complex with *cdtB*, with the *pltB* gene having no apparent effect on cellular distension. However, when *cdtB* is combined with *pltA*, it induces noticeable distension in both the cytoplasm and the nucleus (Mezal et al., 2014). In *S*. Javiana, *cdtB* may require *PltA* for the typical signs of cytolethal distension, such as cytoplasmic and nuclear swelling. The *cdtB* gene, in conjunction with *pltA* (pertussis-like toxin A) and *pltB* (pertussis-like toxin B), is necessary to induce intoxication signs in eukaryotic cells, such as cellular distension and cell cycle arrest (Mezal et al., 2014; Spanò et al., 2008). In the other hand, *cdtB*, with the assistance of *cdtAC*, is translocated into the nucleus of target cells, where it induces double-strand breaks in DNA, resulting in cell apoptosis (Ohara, 2004; Thakur et al., 2022).

## 5 Conclusion

This study has identified a total of 53 *Salmonella* isolates from 300 samples collected during 2019 to 2022 in Shandong, China. Among the 53 *Salmonella* isolates, seven serotypes were identified wherein the *S.* Enteritidis was found to be most prevalent, followed by *S*. Pullorum and *S.* Typhimurium. WGS analysis revealed that ST11, ST92, and ST19 were the dominant sequence types in *Salmonella* in this study. The findings underscore the importance of epidemiological surveillance in the major agricultural area like Shandong Province. Furthermore, the majority of *Salmonella* isolates exhibited features of multi-drug resistance, highlighting the need for precision controlling strategies against such resistant *Salmonella*.

The majority of *Salmonella* isolates in this study exhibited multidrug resistance, highlighting the need for precise control strategies to address these resistant strains. This underscores the critical role of the One Health concept, which recognizes the interconnectedness of human, animal, and environmental health. The emergence and spread of multidrug-resistant *Salmonella* are influenced by various factors, including human, animal, and environmental elements. Therefore, an integrated approach should not only monitor human health but also coordinate animal health management and environmental surveillance. Such a strategy is essential for curbing the spread of resistant bacteria and mitigating the public health risks posed by multidrug-resistant *Salmonella*.

However, several limitations exist in this study. Firstly, it was region-specific, focusing solely on Shandong Province, which limits the generalizability of the findings. Additionally, the sample size was relatively small, and the study did not include *Salmonella* strains from other animals, such as cattle, sheep, and pigs, which may provide insights into cross-species transmission. Moreover, the impact of the rearing environment on *Salmonella* was not explored. To address these gaps, future research should expand the study to broader regions, investigate cross-species transmission, and examine the influence of environmental factors and farming practices. Incorporating the One Health approach into such studies will be crucial for developing more effective strategies to explore and combat antimicrobial resistance.

## Data Availability

The datasets presented in this study can be found in online repositories. The names of the repository/repositories and accession number(s) can be found at: https://submit.ncbi.nlm.nih.gov/subs/, Bioproject ID PRJNA1182193.
